# Regional Heterogeneity in Murine Lung Fibroblasts from Normal Mice or Mice Exposed Once to Cigarette Smoke

**DOI:** 10.1371/journal.pone.0039761

**Published:** 2012-06-28

**Authors:** Olena Preobrazhenska, Joanne L. Wright, Andrew Churg

**Affiliations:** Department of Pathology, University of British Columbia, Vancouver, British Columbia, Canada; University of Tübingen, Germany

## Abstract

Chronic obstructive lung disease (COPD) is characterized by matrix deposition in the small airways but matrix loss from the parenchyma, phenomena which must depend on the ability of local fibroblasts to produce matrix after smoke exposure. To investigate this idea, we exposed C57Bl/6 mice once to cigarette smoke or to air (control) and prepared primary cultures of lung fibroblasts by microdissecting large airways (trachea, LAF), medium size airways (major bronchi, MAF) and parenchyma (PF). Control PF showed the lowest rate of wound closure and wound closure was depressed in all lines by a single *in vivo* smoke exposure. Gene expression of matrix proteins differed considerably among the sites; decorin, which may sequester TGFβ, was markedly higher in PF. PF showed higher intrinsic ratios of pSmad2/Smad2. Smoke caused much greater increases in secreted and matrix deposited collagens 1 and 3 in PF than in LAF or MAF. Expression of Thy-1, a gene that suppresses myofibroblast differentiation, was increased by smoke in PF. We conclude that there is considerable regional heterogeneity in murine lung fibroblasts in terms of matrix production, either basally or after *in vivo* smoke exposure; that PF have lower ability to repair wounds and higher intrinsic TGFβ signaling; and that a single exposure to smoke produces lasting changes in the pattern of matrix production and wound repair, changes that may be mediated in part by smoke-induced release of TGFβ. However, PF still retain the ability to repair by producing new matrix after a single *in vivo* smoke exposure.

## Introduction

Both small airway remodeling and emphysema contribute to airflow obstruction in cigarette smoke-induced COPD, but the morphologic changes that lead to airflow obstruction are diametrically opposite. In the emphysematous parenchyma matrix is lost, whereas in small airway remodeling the bronchioles develop thick fibrotic walls. Since the small airways and parenchyma are, anatomically, connected to each other, these differences in pathologic changes occur over minute distances, implying that they are mediated by intrinsic differences in the small airways and parenchyma.

Production and deposition of matrix is generally a function of fibroblasts/myofibroblasts (reviewed in [1]), and a few papers have suggested that there are regional differences in the properties of fibroblasts in different portions of the human lung ([2]–[6] and see Discussion). Such differences might be important in explaining the response to smoke in the airways and parenchyma, but little is known about this question. In addition, there is evidence that parenchymal fibroblasts from COPD patients differ from those of non-COPD patients in terms of proliferative ability and production of some matrix components (reviewed in [7]), and see Discussion], but there do not appear to be published studies specifically comparing the properties of airway and parenchymal fibroblasts in COPD.

Here we have asked whether there are differences in fibroblast wound healing and production of matrix proteins and factors that control production of matrix proteins in murine fibroblasts from very large airways (trachea), medium size airways (major bronchi), and parenchyma, and whether these properties are affected by a single *in vivo* exposure to cigarette smoke.

## Materials and Methods

### Animals and Smoke Exposure

3 month old female C57BL/6 mice were purchased from Charles River (Montreal, Quebec). Experimental procedures were approved by the University of British Columbia Animal Care Committee. Newly purchased animals were allowed to adjust for 1 week and then divided into control (air exposure) or smoke exposure groups; smoke exposure consisted of four 2R1 Kentucky Research Cigarettes, administered once, using a nose only smoking system previously described [8]. Animals were sacrificed by CO_2_ inhalation 24 hours after starting smoke exposure.

### Isolation of Primary Fibroblasts and Cell Culture

Using a dissecting microscope, large airways (trachea) and medium sized airways (first 2 generations of bronchi) were dissected free of surrounding tissue and opened longitudinally. Epithelial cells were removed from the lumen of the tracheas with a cell scraper, but the medium sized airways were too small to allow removal of the epithelium. Airways were then cut into 1×1 mm^2^ pieces. Small pieces of distal parenchyma were scraped free of pleura and then used as the parenchymal samples.

After washing in ice cold sterile PBS 3 times, tissue explants were placed into wells of 6 well BD Primaria plates (BD Biosciences, Mississauga, ON, Canada) containing Dulbecco’s modified Eagle’s Medium with L-glutamine (GIBCO/Invitrogen, Burlington, ON, Canada) supplemented with 10% Fetal Bovine Serum (GIBCO) and 1% antibiotic/antimycotic solution (GIBCO).

Tissue explants were cultured at 37°C at 5% CO_2_ for 7–10 days with a change of medium every third day. In each experiment the tissue explants for each site from 5 animals were pooled. After reaching confluence cells were split into 75-cm^2^ flasks (BD Bioscience) and referred as passage 1. Primary fibroblasts derived from trachea were designated as large airways fibroblasts (LAF); from bronchi as medium airway fibroblasts (MAF) and from parenchyma as parenchymal Fibroblasts (PF).

Primary cells were plated at a density of 1.25×10^5^ cells/ml for 24 h, then the medium was changed and the cells grown for additional 48 h with or without 2 ng/ml of recombinant human TGFβ1 (R&D Systems, Minneapolis, MN, USA ). Cells between passage number 2–4 were used for all experiments. Each experiment was repeated 2 times with 2 different sets of cells, except as noted, and representative images or average calculations are presented.

### Cell Proliferation Assay

Proliferation of primary fibroblasts was assessed using CellTiter 96 Non-Radioactive Cell Proliferation Assay (Promega, Madison, WI, USA) in accordance with the manufacturer’s protocol. Briefly, equal amount of fibroblasts from different lung regions (4×10^3^ cells) were plated into wells of 96-well plate in full growth medium and let grow for 48 hour, at which time the assay was performed.

### Inhibition of TGFβ Signaling

For inhibition of TGFβ type I receptor (Activin-receptor like kinase 5, Alk5) cells plated as above were treated with the selective Alk-5 inhibitor SB505124 (Sigma-Aldrich).

### Gene Expression

RNA was extracted using RNeasy Mini-Kit (Qiagen, Valencia, CA, USA) as per the manufacturer’s instructions. Reverse transcription of mRNA was carried out by using the High Capacity RNA-to-cDNA kit (Applied Biosystems/Invitrogen). Gene expression was determined by real-time PCR using TaqMan probes and an Applied Biosystems StepOnePlus machine (Applied Biosystems/Invitrogen). The amount of PCR product derived from each mRNA was normalized to that from β-actin in the same sample. The Applied Biosciences TaqMan Probe numbers are: Thy-1: Mm00493681_m1; CTGF: Mm01192931_g1; Collagen 1α1: Mm00801666_g1; Collagen 3α1: Mm01254476_m1; TGFβ1: Mm00441724_m1; Alk5: Mm00436971_m1; TGFβRII: Mm00436977_m1; TGFβRIII: Mm00803538_m1; Fibrillin-1: Mm01334130_m1; ED-A Fibronectin: Mm00692655_m1; Decorin: Mm00514535_m1; Elastin: Mm00514670_m1; Actin: 4352341E.

### Western Blotting

Cells were lysed in buffer containing 50 mM Tris (pH 7.5), 150 mM NaCl, 1% deoxycholic acid, 1% NP40, 0.1% SDS, 2 mM Na_3_VO_4_, 1 mM NaF, 2 mM β-glycerophosphate and “Complete” protease inhibitor cocktail tablets (Roche, Laval, QC, Canada). Protein concentration was measured using a bicinchoninic acid protein assay kit (Pierce/Thermo Fisher Scientific, Rockford, IL,USA) and 10–20 μg of total protein were loaded on SDS-PAGE in 6% or 8% (w/v) Bis-Tris–containing polyacrylamide gels under reducing conditions. Proteins were then transferred to polyvinylidene difluoride membrane (Millipore). Blots were blocked with 5% fat-free milk (Bio-Rad, Mississauga, ON,Canada) in TBS-T (50 mM Tris, 150 mM NaCl, 0.1% Tween-20, pH 7.4) and then incubated with primary antibodies at 4°C overnight. Next, the blots were incubated with horseradish peroxidase–labeled secondary antibody for 1 h at 24°C and then developed with enhanced chemiluminescence Western blotting detection system (Amersham/GE Healthcare, Baie d’Urfe, QC, Canada). Visualization was done with VersaDoc 5000 and Quantity One Software version 4.6.5 (Bio-Rad). Blots were then stripped and reprobed with α-tubulin (Millipore) as a loading control. Quantification was done using Image Lab software version 3.0. (Bio-Rad).

Antibodies used were: Anti-Smad2 (Cell Signaling Technology #3103); anti-phospho-Smad2 **(**Cell Signaling Technology # 3108); anti-E-cadherin (BD Biosciences #610181); anti-vimentin (Millipore #AB1620); anti-CD-45 (Millipore #05-1410); anti-smooth muscle actin (Sigma-Aldrich A 5228); anti-pan-cytokeratin (Santa Cruz sc-81714); anti-α-tubulin (Millipore MAB1864; Anti-Smad4 (Santa-Cruz sc-1909).

### Wound Healing Assay

For the wound healing assay confluent monolayers of cells were scraped with a pipette tip. Culture medium was changed to remove detached and damaged cells and wound closure was monitored microscopically at different time points. The six different cell lines were wounded at the same time in two independent experiments and migration was determined using the ImageJ program as an average closed area of the wound relative to the initial wound area at 16 h after wounding.

### Light and Immunofluorecent Microscopy

Both brightfield and fluorescent images were obtained using a microscope equipped with a CCD camera (Empix Imaging, Inc, Toronto, ON, Canada). Images were analyzed using image analysis software (Northern Eclipse V8, Empix Imaging Inc.). For immunofluorescent microscopy, cells were plated on Lab-Tek Chamber Slides (Nalge Nunc International/Thermo Fisher Scientific, Ottawa, ON, Canada), fixed with 4% paraformaldehyde in phosphate buffered saline (PBS), permeabilzed with 0.2% Triton X-100 in PBS, and stained with mouse monoclonal antibodies directed against SMA and TRITC conjugated Phalloidin (both from Sigma-Aldrich). The detection was done with anti-mouse Alexa Fluor 488 (Molecular Probes/Invitrogen ). The antibody used against smooth muscle actin was purchased from Sigma Aldrich (#5228); TRITC conjugated Phalloidin from Sigma-Aldrich (#77418); and Alexa Fluor 488 from Molecular Probes/Invitrogen (#A11029). Samples were mounted in Vectashield Mounting Medium containing 4′,6-diamidine-2-phenylidole-dihydrochloride (DAPI; Vector Laboratories Inc., Burlingame, CA, USA) according to the manufacturer’s protocol.

**Figure 1:**
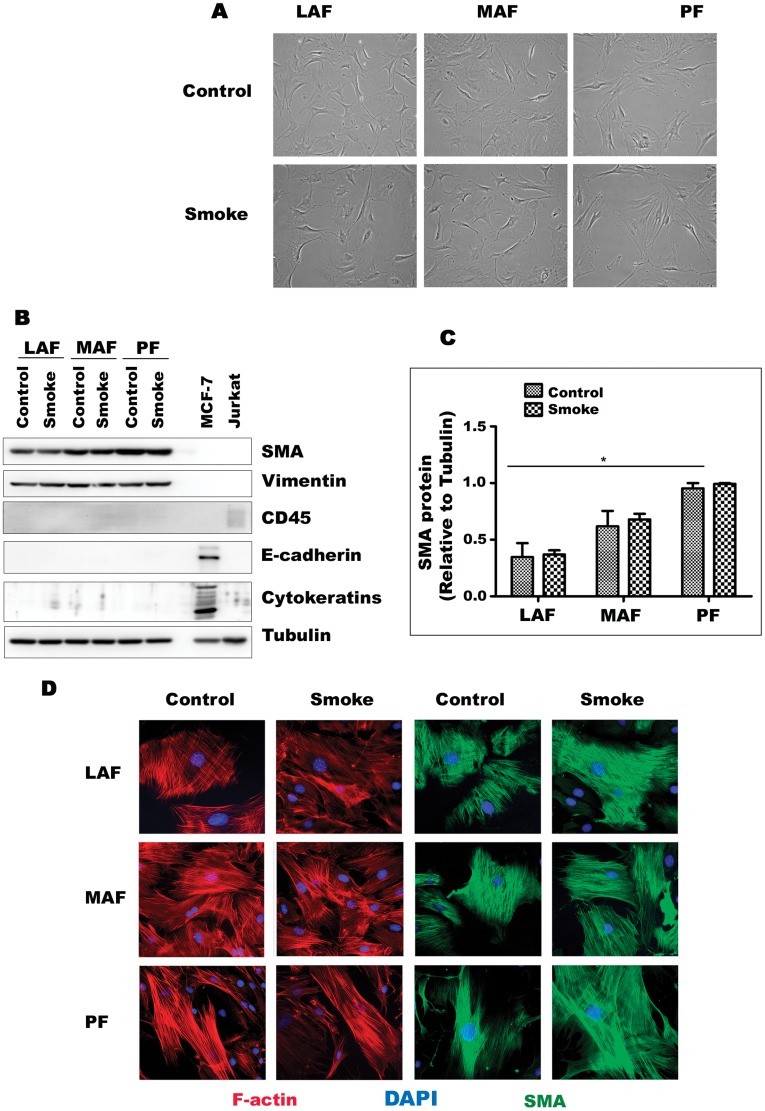
Primary cells from mouse lung display the morphology and phenotype of fibroblasts. A. Appearance of fibroblasts from LAF, MAF, and PF exposed to air (control) or smoke. All are morphologically similar and their appearance is not altered by smoke exposure. B. Representative western blot of lineage markers. All lines express the fibroblast/myofibroblast markers smooth muscle actin (SMA) and vimentin and are negative for the hematopoietic marker CD45, the epithelial markers e-cadherin and cytokeratins. MCF-7 and Jurkat cells are included as positive epithelial and hematopoietic controls. C. Quantification of Western blot results for SMA . Data are mean+/−SD. *P<0.05 D. Immunofluorescent staining for F-actin and SMA showing prominent stress fibers and the typical microfilament array of fibroblasts.

### Production of Extracellular Matrix Proteins

Synthesis, secretion and deposition of collagen 1α1, collagen 3α1 and EDA-Fibronectin were determined by Western blot analysis. For the analysis of secreted proteins aliquots of conditioned medium were taken. The intracellular fraction of protein was extracted by incubating monolayers of cells with 1% Triton X-100 lysis buffer (1% Triton X-100, 150 mM NaCl, 20 mM Tris-HCl pH 7.4 plus protease inhibitor tablets) on ice for 10 min. The Triton-insoluble extracellular matrix bound fraction remaining attached to the plastic was washed twice with ice-cold PBS and solubilized directly in SDS sample buffer. The samples were loaded on a 7% SDS-polyacrylamide gel in proportion to the amount of total protein in cultures. Western blot procedure was carried as described**.** Antibodies used were: anti-collagen 1α1 (Santa Cruz sc-25974); anti-collagen 3α1 (Santa Cruz sc-28888), and anti-EDA-fibronectin (Santa Cruz sc-59826).

### Statistical Analysis

For most experiments the presented data is derived from 2 separate groups of pooled cells with the pool derived from mixed cells of 5 different mice as described above. The wound healing experiments are derived from 6 different wounds per treatment group. Data are expressed as mean+/−SD. Statistical significance was evaluated by ANOVA followed by Turkey multiple comparison tests as appropriate using PRISM software (GraphPad, San Diego, CA, USA). Differences with P<0.05 were taken as statistically significant.

**Figure 2:**
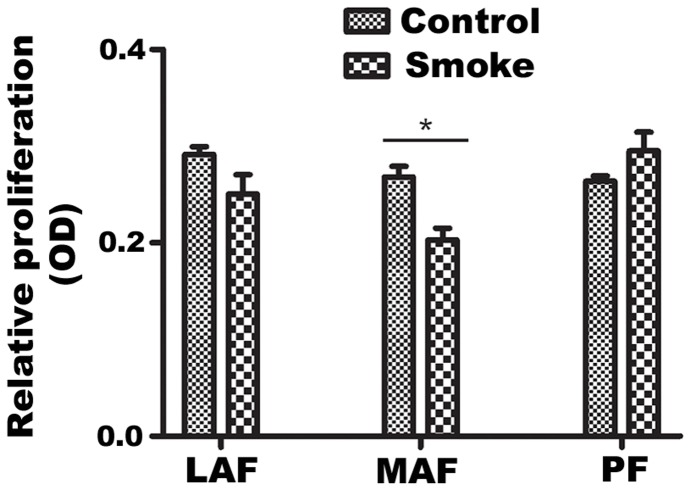
Proliferation of rate of primary fibroblasts. Relative proliferation was evaluated using the MTT assay, as described in Materials and Methods. LAF, MAF and PF demonstrated similar proliferation rates. A single exposure to smoke had no effect on LAF and PF proliferation, but slightly decreased growth of MAF. Data are mean+/−SD. *P<0.05, **P<0.01, ***P<0.001.

## Results

### Cell Morphology and Lineage

Cells isolated from large airway, small airway, and parenchymal samples displayed typical fibroblast morphology (Fig. 1A) and there were no obvious differences in cell shape or size by site of origin. Western blot showed that all lines expressed smooth muscle actin and vimentin and did not express the epithelial markers cytokeratins or E-cadherin, or the hematopoietic cell marker CD45 (Fig. 1B). Baseline levels of smooth muscle actin increased from LAF to MAF to PFs. Cytoskeleton organization in all cell lines also was typical for fibroblasts with pronounced stress fibers and actin microfilament network (Fig. 1D). Cell lines derived from animals exposed once to cigarette smoke did not show obvious morphologic differences or major differences in production of smooth muscle actin and vimentin compared to cells derived from air-exposed controls (Fig. 1).

**Figure 3:**
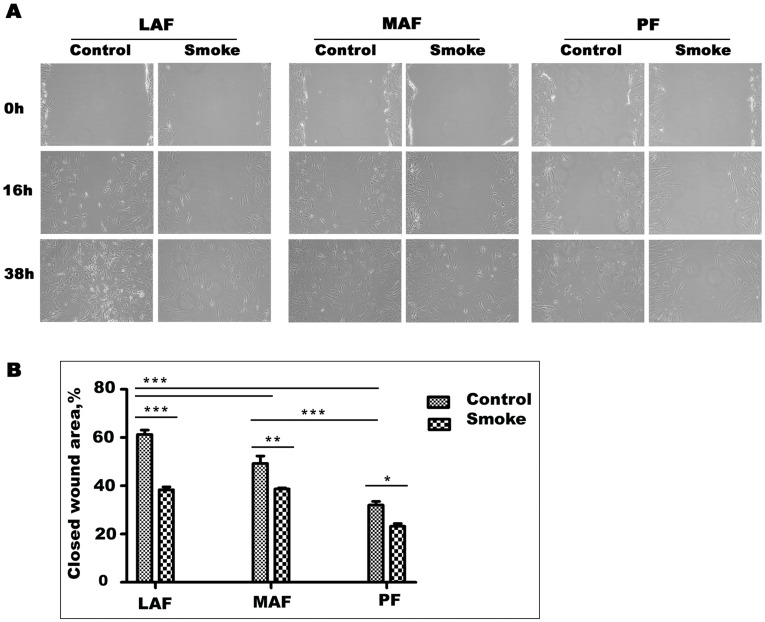
Wound healing assay. A. Representative wound healing images at 0, 16, and 38 hours. Wounds were made with a pipette tip in confluent monolayers. Wound healing rates decrease from LAF to MAF to PF and are decreased by smoke exposure in cells from each site. B. Quantification of wound healing rates. Data are mean+/−SD. *P<0.05, **P<0.01, ***P<0.001.

### Cell Proliferation

Control LAF, MAF and PF displayed similar proliferation rates (Fig. 2). A single exposure of animals to smoke did not affect relative proliferation in LAF and PF, and caused a slight, but statistically significant decrease in MAF.

**Figure 4:**
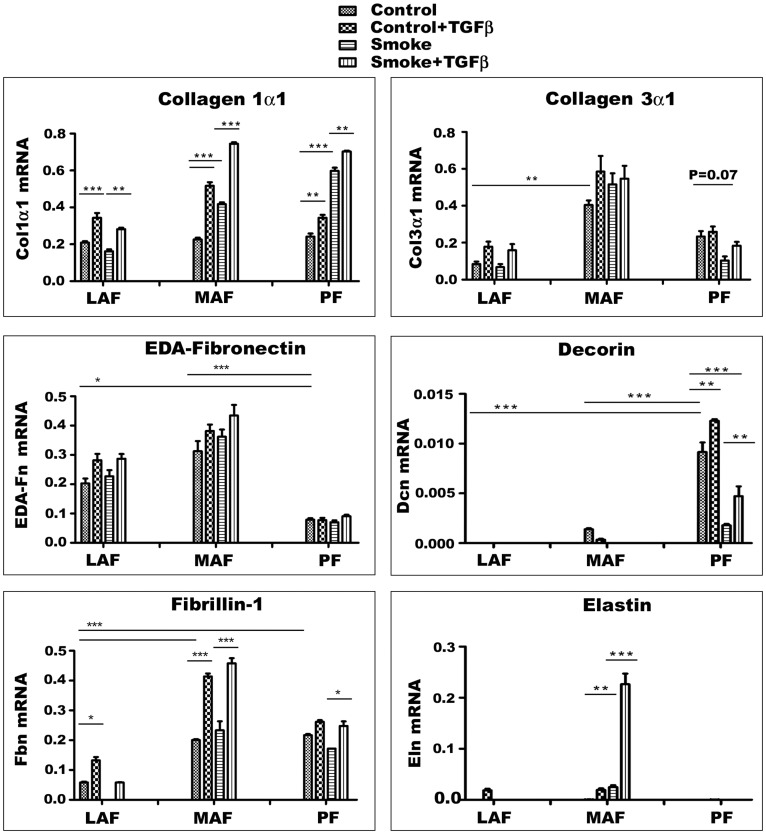
Expression levels of genes for matrix proteins. Cells are derived from air (Control) or smoke exposed (Smoke) animals and some have been exposed to exogenous TGFβ1 *in vitro.* Gene expression varies considerably among the three sites. Decorin is the only gene which is much more highly expressed in PF in the basal state. Smoke increases expression of procollagen 1α1 and decreases expression of decorin. Although there is site to site and gene to gene variability, exogenous TGFβ1 has relatively little effect (except for procollagen 1α1 and fibrillin-1 in MAF, and the combination of smoke plus TGFβ1 on elastin expression in MAF) suggesting that PF in particular normally have high intrinsic TGFβ signaling. Overall, these data show that PF can repair by producing matrix molecules after a single *in vivo* smoke exposure. *P<0.05, **P<0.01, ***P<0.001. Data are mean+/−SD.

### Wound Healing

Wound healing may be important in repair of smoke-induced damage and wound healing assays also provide an indication of cell migration rates. As shown in Fig. 3, basal wound healing rates progressively decreased from LAF to MAF to PF, with the latter showing about 50% of the rate of LAF at 16 hours . Cells from smoke-exposed animals closed wounds significantly slower than those from air-exposed animals at all sites, although the differences from control cells were not marked (Fig. 3B).

### Expression Profile of Matrix-related Genes

Cells derived from the 3 different anatomic sites showed different patterns of gene expression (Fig. 4). Basal procollagen 1α1 expression was similar in LAF, MAF, and PF, and was unchanged by smoke exposure in LAF, whereas in MAF and PF expression was roughly doubled. For procollagen 3α1 (Col3α1) basal expression was greater in MAF compared to LAF or PF. Smoke had no significant effect on Col3α1 expression except for parenchymal fibroblasts where it caused about a 60% decrease in PF (p = .07). EDA-Fibronectin (EDA-Fn), a molecule linked to myofibroblastic differentiation of fibroblasts, was expressed at higher levels in LAF and MAF compared to PF. Decorin is believed to be involved in TGFβ binding (see Discussion) and expression was highest in PF and lowest in LAF and smoke markedly decreased decorin expression, particularly in PF. Expression of fibrillin-1, a protein necessary for proper elastin assembly [9], [10] was higher in MAF and PF compared to LAF and was unchanged by smoke exposure. Tropoelastin, the precursor of elastin, a molecular which is necessary for proper mechanical properties of the alveolar wall, was expressed at extremely low levels in control fibroblasts from all 3 sample sites and was significantly increased by smoke only in MAF.

The effects of added TGFβ1 are also shown in Fig. 4. In general the effect of added TGFβ was to increase expression of matrix proteins, but for most genes from most sites the effects were small. However, TGFβ1 roughly doubled expression of procollagen 1α1 in LAF and MAF but not in PF; fibrillin-1 expression was increased in MAF by TGFβ1. The combination of smoke plus TGFβ1 produced a dramatic increase in expression of tropoelastin in MAF.

**Figure 5:**
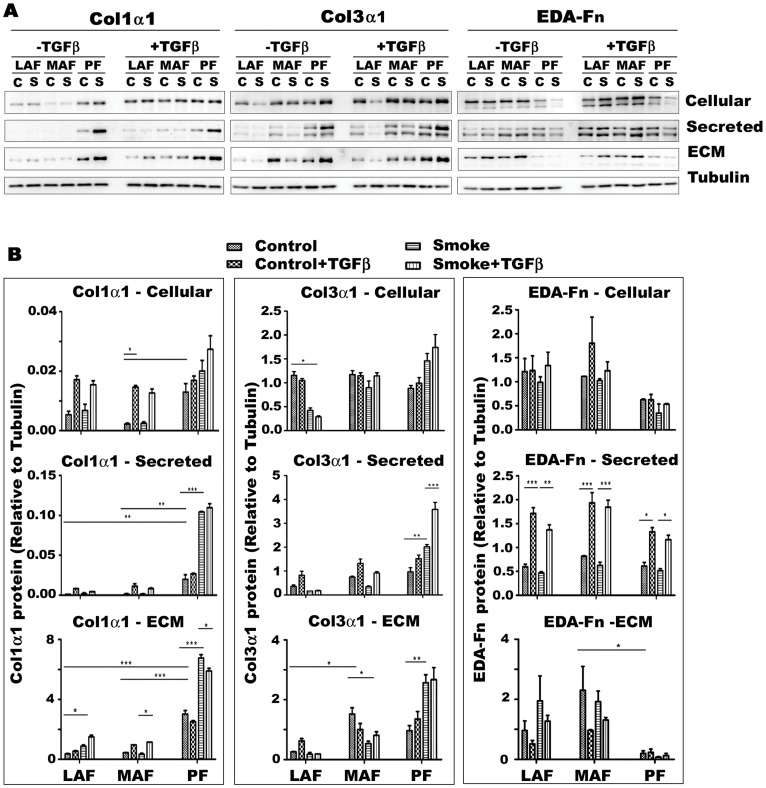
Production and deposition of extracellular matrix proteins by primary fibroblasts from different sites of lung. Representative western blots (A) and quantification (B) of cellular, secreted and extracellular matrix bound (ECM) collagen 1α1 (Co1α1), collagen 3α1 (Col3α1) and EDA-Fn in cells from control (air exposed, C) or smoke (S) exposed animals. Basal levels of Col1α1 are higher in PF and are increased by smoke exposure but not by exogenous TGFβ1 in PF; however exogenous TGFβ1 increases Col1α1 production in LAF and MAF. Cigarette smoke tends to decrease production of Col3α1 in MAF and LAF and increase production in PF. Extracellular matrix bound EDA-Fn is considerably lower in PF. Smoke has little effect on production of EDA-Fn, but the secreted protein is markedly increased by exogenous TGFβ1 in cells from all 3 sites. Data are mean+/−SD. *P<0.05, **P<0.01, ***P<0.001.

**Figure 6:**
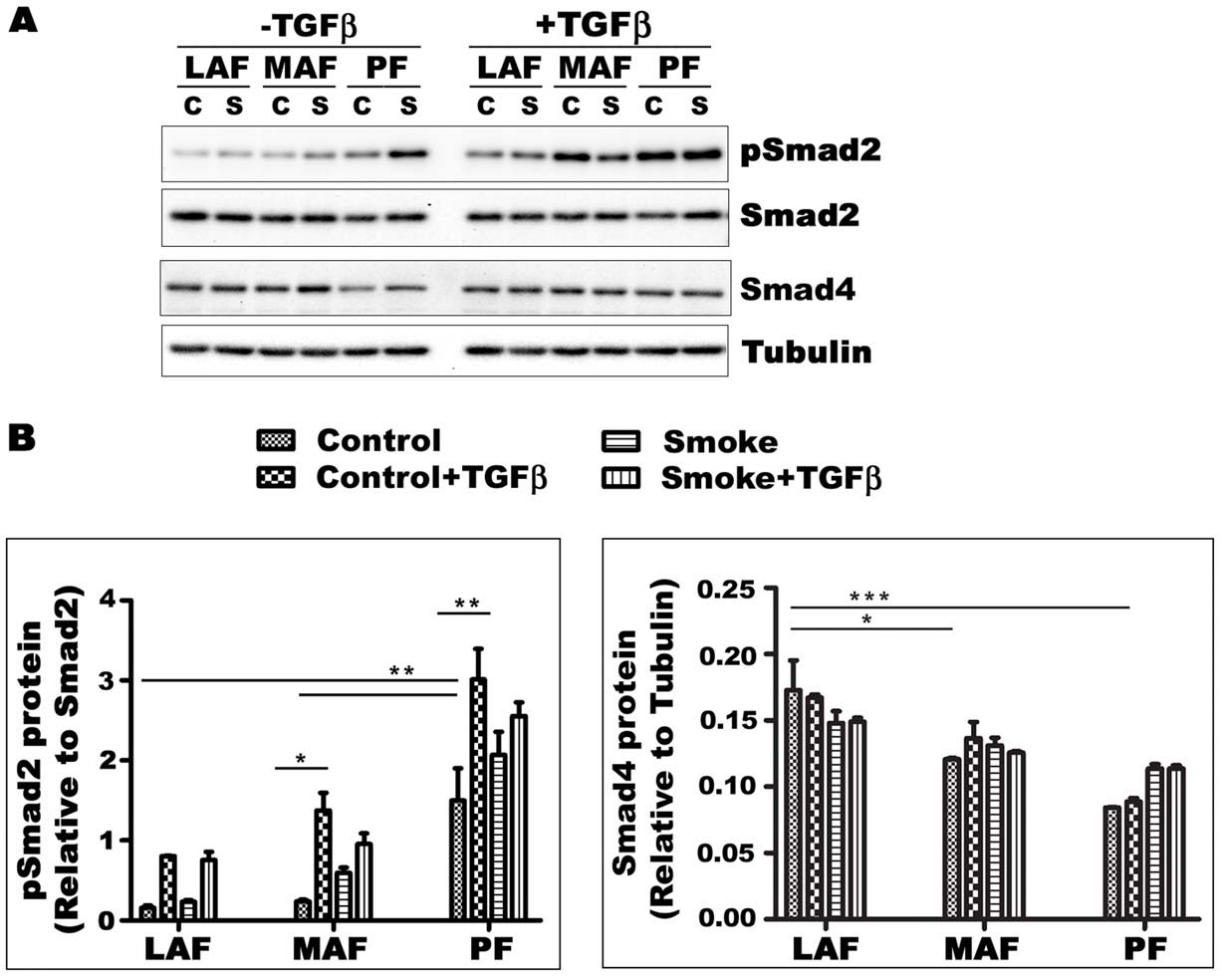
TGFβ signaling in primary mouse fibroblasts from different sites of lung. A. Expression of phosphorylated and nonphosphorylated Smad2 protein, as a measure of TGFβ signaling, and Smad4 in primary fibroblasts. B. Densitometry for pSmad2/Smad2 and Smad4. Ratios of pSmad2/Smad2 are higher in PF compared to LAF and MAF and are increased by cigarette smoke as well as exogenous TGFβ. The findings suggest that PF have higher levels of intrinsic TGFβ signaling and that smoke leads to TGFβ release. C-Control - fibroblasts from animals exposed to air; S- Smoke - fibroblasts from animals exposed to cigarette smoke. Data are mean+/−SD.

**Figure 7:**
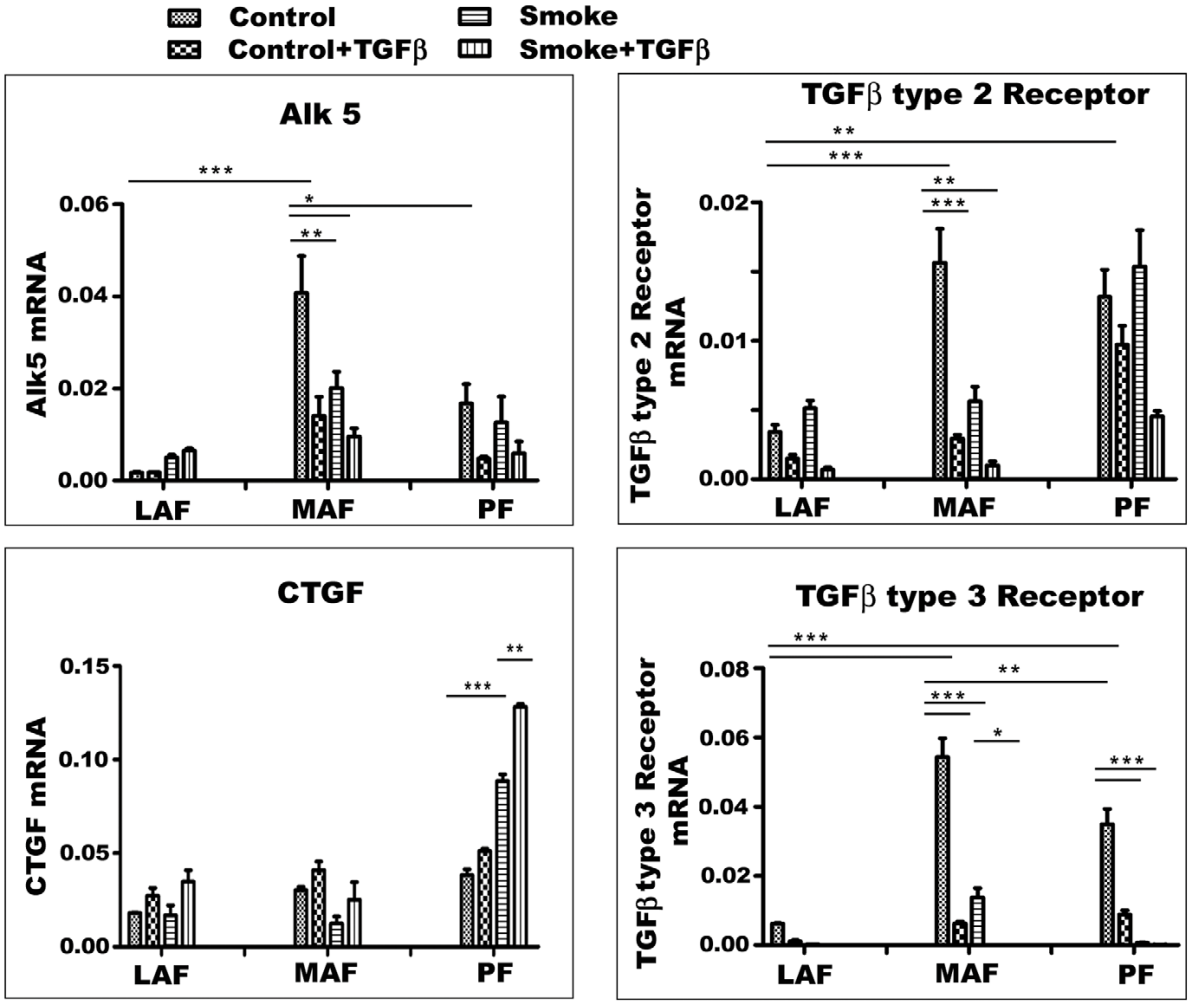
Expression of TGFβ signaling related genes. Gene expression for CTGF, a downstream mediator of TGFβ signaling, and for TGFβ receptors 1 (Alk5), 2, and 3. Smoke increases CTGF expression in PF, suggesting that smoke acts to release TGFβ. TGFβ receptor gene expression is quite variable but tends to be highest in MAF. Smoke and exogenous TGFβ tend to decrease expression of all 3 receptors. Data are mean+/−SD. *P<0.05, **P<0.01, ***P<0.001.

**Figure 8:**
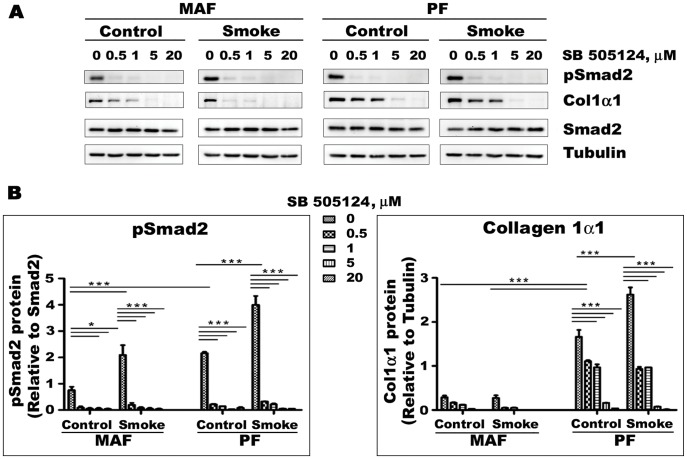
Effects of the Alk5 inhibitor SB505124 on pSmad2/Smad2 ratios and Collagen 1α1 production in MAF and PF. A- Representative Western blot image. B- Quantification of Western blot results for pSmad2 and Col1α1. pSmad2/Smad2 ratios and collagen 1α1 production are higher in PF and with smoke exposure, and SB505124 decreases these values, indicating that PF have higher intrinsic TGFβ signaling and that smoke causes TGFβ release. Data are mean+/−SD. *P<0.05, **P<0.01, ***P<0.001.

### Levels of Matrix Proteins

Collagen 1α1 (Col1α1) protein was produced in considerably greater quantities by PF compared to MAF or LAF (Fig. 5), and this was true whether cell lysates, supernatants, or extra-cellular matrix was examined. Smoke exposure markedly increased supernatant and matrix bound Col1α1 production by PF but not MAF or LAF. Basal Col3α1 protein showed a variable pattern, with larger amounts of protein in the extracellular matrix produced by MAF and PF; cigarette smoke increased Col3α1 production by PF and decreased it in MAF. Basal levels of matrix EDA-Fn were highest in LAF and MAF and considerably lower in the matrix in PF and smoke produced only small changes.

Exogenous TGFβ markedly increased Col1α1 production by LAF and MAF but had no effect on PF. Exogenous TGFβ had only very small effects on Col3α1 production. The effect of exogenous TGFβ was to generally increase secretion of EDA-Fn into the supernatant and decrease matrix deposition of EDA-Fn.

**Figure 9:**
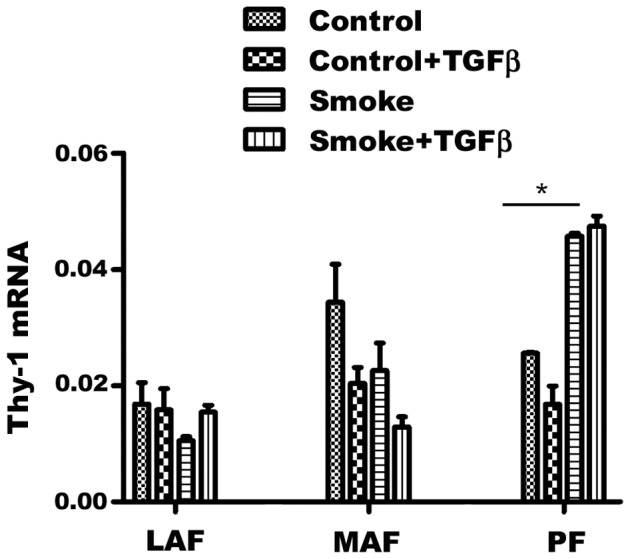
Gene expression of Thy-1. Thy-1 is involved in control of fibroblast to myofibroblast differentiation. Basal levels are not statistically different among the 3 sample sites, but cigarette smoke tends to drive expression down in MAF and up in PF, a process that might eventually lead to failure of PF to produce matrix (see text). Control - fibroblasts from animals exposed to air; Smoke – fibroblasts from animals exposed to cigarette smoke. Data are mean+/−SD. *P<0.05.

### TGFβ Signaling

Since TGFβ is an important driver of matrix production, we examined TGFβ signaling pathways (Figs. 6, 7, 8). We tried to demonstrate Smad3 using several different commercial antibodies, and, although we were readily able to detect a signal in fetal lung fibroblasts, we could not detect Smad3 in our any of our lines. For that reason, Smad2 and phopho-Smad2 (pSmad2) were evaluated instead and analyzed as the ratio of pSmad2/Smad2. In cells from air-exposed animals, this ratio was similar in LAF and MAF and was 2 to 3 times higher in PF (Fig. 6). Smoke exposure increased the pSmad2/Smad2 ratio in MAF and PF. Smad4 levels were lower in PF compared to LAF and were not significantly affected by smoke or exogenous TGFβ in fibroblasts from all three sites (Fig. 6). CTGF is believed to be a proximate mediator of TGFβ-induced collagen production. Basal levels of CTGF gene expression were not significantly different in the 3 sample sites but were decreased by smoke in MAF and increased about 2 fold in PF (Fig. 7). Taken in aggregate these findings suggest that PF show higher levels of intrinsic TGFβ signaling compared to LAF and MAF and that smoke increases TGFβ signaling. Addition of exogenous TGFβ increased the pSmad2/Smad2 ratio but had relatively little effect on CTGF expression.

Fig. 7 also shows TGFβ receptor gene expression patterns. Type 1 (Alk 5) and 3 receptor basal expression were higher in MAF compared to LAF and PF whereas type 2 receptor expression was highest in MAF and PF, and in general both smoke and exogenous TGFβ decreased expression of these genes.

To further examine the role of TGFβ, we blocked TGFβ signaling using the Alk5 inhibitor SB505124 (Fig. 8); only MAF and PF were used for these experiments since these compartments are the ones primarily affected by cigarette smoke. SB505124 decreased the pSmad2/Smad2 ratio and inhibited Col1α1 production in a dose response fashion. These findings also suggest that there is a high level of intrinsic TGFβ signaling in lung fibroblasts.

### Control of Fibroblast to Myofibroblast Transformation

Myofibroblasts typically exhibit smooth muscle actin production, and, as shown in Fig. 1, smooth muscle actin levels increased from LAF to MAF to PF but were not affected by smoke exposure. Thy-1 is a gene that, when expressed, prevents differentiation of fibroblasts to myofibroblasts ([1] and see Discussion); in our cell lines basal Thy-1 expression was decreased by about 40% in MAF by smoke and upregulated about 2 fold in PF (Fig. 9 and see Discussion).

## Discussion

The idea that the lung exhibits abnormal repair in COPD has become a subject of increasing interest, and it has been proposed that a fundamental cause of emphysema is a failure to repair smoke-induced damage (reviewed in [7]); more specifically it has been suggested that the inability of the parenchyma to repair may result from relative TGFβ insensitivity [7]. While this concept is intriguing, it has not been looked in terms of the differences between parenchymal and airway fibroblasts.

The concept of regional fibroblast heterogeneity as the basis of local differences in reaction pattern/anatomic changes in lung disease is an attractive idea that has been little explored. Older morphologic studies have demonstrated that lung parenchymal fibroblasts stain for smooth muscle actin and are contractile, supporting the idea that they are myofibroblasts, but only a few studies have examined this issue in more detail.

Sugiura et al [6] used cultured large airway and parenchymal fibroblasts from an ovalbumin asthma model in Balb/c mice and looked at phenotypes related to repair and remodeling. Although the fibroblasts from the ovalbumin challenged animals showed increased proliferation, VEGF, fibronectin, TGFβ1, and smooth muscle actin production compared to control fibroblasts, there were essentially no differences between large airway and parenchymal fibroblasts.

Nihlberg et al [5] examined matrix production in fibroblasts cultured from distal and central airways in control and human mildly asthmatic subjects and found that distal control fibroblasts proliferated faster and had more extensions than central fibroblasts. Distal control fibroblasts produced more versican than central fibroblasts but no differences were seen in production of biglcan, perlecan, and decorin. Fibroblasts from the asthmatic patients proliferated more slowly than those from the controls. Kotaru et al [2] cultured fibroblasts from proximal airways and distal parenchyma of 13 asthmatic and 2 non-diseased autopsy subjects. They found that asthmatic airway fibroblasts were larger and more stellate than parenchymal fibroblasts and synthesized more procollagen type 1 and eotaxin, while distal fibroblasts proliferated much faster and expressed more smooth muscle actin; however similar site-related phenomena were seen in the fibroblasts from the normal autopsy lungs. The authors concluded that lung fibroblasts from different sites are phenotypically different. More recently the same group [3] performed microarray analysis on 12 matched pairs of airway and parenchymal fibroblasts and found that parenchymal fibroblasts showed evidence of enhanced TGFβ signaling, and increases in genes associated with cytoskeletal regulation whereas airway fibroblasts had increased expression of genes controlling extracellular matrix organization. In both sets of experiments there were no differences between asthmatic and normal fibroblasts.

Pechovsky et al [4] similarly examined proximal bronchial and parenchymal fibroblasts from 12 human subjects (7 non-smokers with no lung disease and 5 with lung cancer). Subject- matched parenchymal fibroblasts were found to have higher levels of smooth muscle actin as well as intrinsic autocrine TGFβ signaling reflected in high phospho-Smad3/Smad3 levels and increased cell stiffness, a finding that they suggested related to TGFβ activation via integrins α_v_β_5_ and α_v_β_3_. However, bronchial fibroblasts expressed higher levels of CTGF, fibrillin 1, and fibronectin 1 and there were no differences in procollagen 1 or 3 expression.

The different protocols employed and end points examined as well as the different underlying lung conditions make it difficult to arrive at a consistent set of conclusions from these various experiments. However, two things that do emerge are the idea that local fibroblast differences are phenotypically stable since they persist through multiple passages *in vitro*, and, of particular interest, the finding that an environmental challenge such as ovabumin sensitization can lead to a either a persisting change in fibroblast phenotype or selective expansion of a clone of fibroblasts with a particular phenotype.

In this paper we have looked at intrinsic fibroblast differences and the effects of cigarette smoke and exogenous TGFβ in 3 different locations: large airways (trachea), medium sized airways, and parenchyma. As indicated above, all of the published data on fibroblast heterogeneity have used human airway (bronchial) samples, presumably for technical reasons, but in humans with cigarette smoke-induced COPD and in animal models of cigarette smoke-induced COPD, it is the small airways (bronchioles) that become fibrotic and narrowed. Ideally we would like to be able to culture fibroblasts from bronchioles, but in the mouse these are physically very small, and, although we are able to microdissect down to the bronchiolar level, we have not been successful in culturing fibroblasts from such dissections.

It is clear that there are considerable differences in the phenotype of fibroblasts cultured from these 3 different sites. Like Kotaru et al [2] and Pechkovsky et al [4], we observed that parenchymal fibroblasts exhibited higher intrinsic levels of smooth muscle actin, generally regarded as a marker of myofibroblast differentiation. Wound healing, which is in part a measure of cell migration, decreases as one moves distally in the lung. In the basal state, expression of matrix related genes tends to be highest in MAF with the exception of decorin which is much higher in PF; these findings are similar to the observations of Pechkovsky et al [4] on human fibroblasts. However, translation is clearly different from transcription for many of these genes, since the production of collagen proteins and particularly matrix bound collagens by our murine cells is at least as high in PF as MAF and for Col1α1 is higher.

Also, similar to the Pechkovsky study, we observed that PF show higher levels of intrinsic TGFβ signaling, visible both as higher intrinsic pSmad2/Smad2 ratios and in the generally small response of PF to exogenous TGFβ1, implying that they are already TGFβ-stimulated. These observations also show that PF are perfectly capable of producing matrix proteins after a single smoke exposure; ie, failure to repair the parenchyma after smoke exposure does not reflect an intrinsic problem with the ability of parenchymal fibroblasts to repair but must likely reflects an acquired change in phenotype.

This idea is supported by finding that even a single *in vivo* smoke exposure produces persisting changes in fibroblast phenotype in all 3 sites examined. This is clearly reflected in the pattern of matrix gene expression and collagen protein production; the latter is increased in PF. On the other hand, expression of decorin and wound repair is decreased in all sites. Decorin production may be important in controlling TGFβ driven processes (see below). Overall, however, PF are still capable of repair reactions after a single smoke exposure. In contrast to PF, in MAF, the site of small airway remodeling, the changes in collagen production in PF after smoke exposure lead to an increase in the ratio of collagen 1 to collagen 3, an effect seen in scar formation in general.

Cigarette smoke is a concentrated source of oxygen and nitrogen radicals, and we have previously shown that smoke can oxidatively activate latent TGFβ in tracheal explant cultures [11]. To further examine this idea, we treated our current cultures with the Alk5 inhibitor SB505124, and found that SB505124 decreased basal pSmad2/Smad2 ratios and Col1α1 production, lending support to the idea that basal fibroblast matrix production in the lung is driven by endogenous TGFβ**** signaling. SB505124 also prevented smoke mediated increases in collagen production in PF,suggesting that smoke-induced release of TGFβ plays a major role in determining the response of PF.

In our cultures basal decorin expression is much higher in PF compared to airway fibroblasts. Decorin is a matrix proteoglycan that appears to play a role in mediating TGFβ signaling by binding TGFβ and sequestering it in the matrix [12]–[14], and transient expression of decorin using an adenoviral vector reduces bleomycin-induced fibrosis [15]. The dramatic decrease in decorin gene expression in PF from smoke-exposed animals might also be important in allowing TGFβ release. Decreased sequestration of TGFβ by decorin could be another mechanism by which smoke upregulates production of some matrix components [16].

Thy-1 is a membrane glycoprotein that, when expressed, tends to downregulate myofibroblastic features, production of matrix, and contractility in fibroblasts [1], [17]; conversely, loss of Thy-1 expression increases the resistance of fibroblasts/myofibroblasts to apoptosis and increases fibrosis, for example, in the fibroblast foci of usual interstitial pneumonia [18]. In the present study MAF and PF show opposite patterns of Thy-1 gene expression after smoke exposure, with a tendency to decrease in MAF and an increase in PF. While the overall difference in expression levels between MAF and PF is not great (about 2 fold after a single smoke exposure), this difference, if maintained with chronic smoke exposure, might slowly shut down the ability of PF to repair smoke-induced matrix damage.

In summary, there are significant differences in the patterns of matrix production, patterns of TGFβ signaling, and in the response to smoke among LAF, MAF, and PF, and a single *in vivo* smoke exposure can producing persisting phenotypic changes. PF have higher intrinsic TGFβ signaling, and smoke exposure appears to release TGFβ, as we have previously suggested [11]. PF are still able to repair by producing new matrix after a single *in vivo* smoke exposure; we are currently investigating whether PF lose this property with long term *in vivo* smoke exposure.
